# A Review of Chronic Lateral Ankle Instability and Emerging Alternative Outcome Monitoring Tools in Patients following Ankle Ligament Reconstruction Surgery

**DOI:** 10.3390/jcm13020442

**Published:** 2024-01-13

**Authors:** Ibrahim Saliba, Alexandre Hardy, Wenzheng Wang, Raphael Vialle, Sylvain Feruglio

**Affiliations:** 1 LIP6 Department, Sorbonne Université, 75005 Paris, France; wenzheng.wang@lip6.fr (W.W.); sylvain.feruglio@lip6.fr (S.F.); 2 Clinique Du Sport, 75005 Paris, France; alexandre.hardy@me.com; 3 Armand-Trousseau Hospital, 75012 Paris, France; raphael.vialle@aphp.fr

**Keywords:** ankle instability, lateral ankle instability, chronic ankle instability, diagnostics, postoperative management, rehabilitation, NIRS, mechanical impedance and bioelectrical impedance

## Abstract

Ankle sprains are exceedingly common injuries in both athletes and the general population. They account for 10 to 30% of all sports injuries. Although the vast majority of lateral ankle ligament injuries respond successfully to conservative management, the absolute number of those that progress to chronic lateral ankle instability (CLAI) remains considerably important. This condition is characterized by persistent symptoms and may be associated with short-term and long-term complications and functional deficits. There is still a lack of ideal postoperative management of CLAI patients. Furthermore, an evidence-based rehabilitation phasing does not exist and most of the published studies regarding this subject suggest some protocols based on a wide variety of functional assessment scores and other modalities that are not accurate enough. Moreover, the literature that assesses the ability to return to work (RTW) and return to sport (RTS) in the general population and athletes operated for CLAI most commonly shows aggregated results with global rates of RTW or RTS without describing a detailed timeline based on the readiness of patients to return to each level of activity. Although stress radiographs and MRI have been assessed as potential tools to improve postoperative management of CLAI patients, the first modality is limited by its low sensitivity to detect laxity and the second one by its static character and its inability to predict neither the healing process phase nor the mechanical properties of the repaired/reconstructed ligaments. Bioelectrical impedance, mechanical impedance and near-infrared spectroscopy are non-invasive methods of measurement that could be potential assessment tools to help surgeons improve the postoperative management of patients after CLAI surgery.

## 1. Introduction

Ankle sprains are exceedingly common injuries in both athletes and the general population [1,2,3,4]. They account for 10% to 30% of all sports injuries [5]. Inversion-type is the most common mechanism of injury, occurring in 80 to 90% of cases [3,5]. Although the vast majority of lateral ankle ligament injuries respond successfully to conservative management, 10% to 30% of patients may develop chronic lateral ankle instability (CLAI) [1,6]. This condition is characterized by persistent symptoms including pain, feeling of insecurity and a sensation of giving way. Moreover, it may result in short and long-term functional deficits, with risk to other structures requiring surgical correction including osteochondral injuries and peroneal tendons pathology [5,7]. There is a variety of surgical procedures intended to address CLAI, ranging from ligament repair to reconstructions using auto- or allograft tissue in order to replace torn ligaments [2,5,8,9,10]. Currently, there is still a lack of uniform phasing or even individualized protocols for rehabilitation of patients following CLAI surgery regarding weight bearing, Return To Work (RTW) and Return To Sport (RTS) [3,11]. As shown by Clements et al. in their systematic review [11], the postoperative outcomes in most studies are being measured using a variety of objective and subjective functional assessment scores and questionnaires, Range Of Motion (ROM) on physical examination and stress radiographs. However, these outcome measurement methods do not reflect the histologic stage of the healing process of the repaired/reconstructed ligaments nor the mechanical stability of the ankle joint, except for stress radiographs, which are limited by their low sensitivity [2,7,12,13]. The aim of this article is to review and assemble the most important and recent literature on CLAI and to suggest possible methods that can guide the surgeons in the future to better manage CLAI patients postoperatively by providing precise data concerning the healing process of tissue and the stability of ankle joint reflecting the ability to RTS.

## 2. Methods

This narrative review was realized by conducting a systematic literature search using the keywords “ankle instability”, “lateral ankle instability”, “chronic ankle instability”, “diagnosis”, “management”, “surgical treatment”, “medical treatment”, “postoperative management”, “postoperative rehabilitation”, “Near Infrared Spectroscopy (NIRS)”, “mechanical impedance” and “bioelectrical impedance”. These terms were queried both independently and in various combinations across the Medline and Embase databases spanning from their earliest possible entry to February 2023. Identification and initial screening of pertinent studies were carried out based on their titles and abstracts. Only articles written in English were included. Exclusion criteria encompassed studies lacking abstracts and those related to management and treatment strategies that lacked reported clinical outcomes or were published over a decade prior to the research conducted between January 2023 and February 2023.

## 3. Overview of Ankle Lateral Ligamentous Anatomy

**Figure 1:**
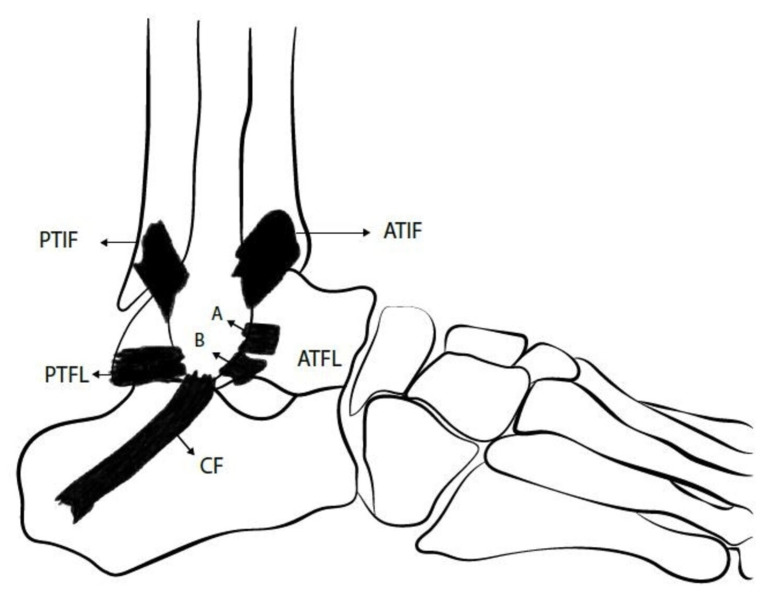
This figure represents the lateral ankle ligament anatomy: superior (A) and inferior (B) bands of Anterior TaloFibular Ligament (ATFL), CalcaneoFibular (CF) ligament and Posterior TaloFibular Ligament (PTFL). ATIF = Anterior inferior TibioFibular ligament; PTIF = Posterior inferior TibioFibular ligament (This figure was created by the authors to be used in this paper).

As shown in Figure 1, three ligaments compose the lateral ligament complex of the ankle: Anterior TaloFibular Ligament (ATFL), Posterior TaloFibular Ligament (PTFL) and CalcaneoFibular Ligament (CFL) [5,14]. ATFL is formed by superior intra-articular and inferior extra-articular fascicles. The inferior fascicle shares its fibular insertion with the CFL and both are connected together by the arciform fibers. Thus, they are sometimes also referred to as the lateral fibulotalocalcaneal ligament complex [5,15,16].

Given the aforementioned findings, the ATFL provides restraint to talar translation in the sagittal plane as well as talar rotation in the axial plane, while the CFL controls talar and calcaneal inversion [2]. Moreover, evidence from biomechanical studies shows that CFL contributes to subtalar joint stability, but the talocalcaneal ligaments do not provide stability to the ankle joint [2,17,18]. It was also observed that maximal tensile force of ATFL occurs during ankle plantar flexion while for CFL, the maximal tensile force occurs during ankle dorsiflexion [2,19].

## 4. Mechanism of Injury

**Figure 2:**
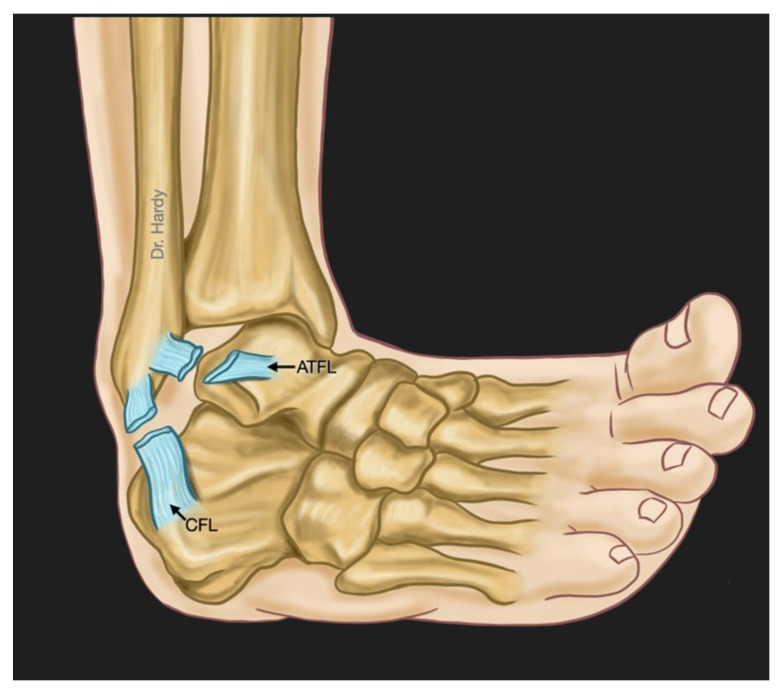
This figure shows a combined rupture of both Anterior TaloFibular Ligament (ATFL) and CalcaneoFibular Ligament (CFL) during an inversion-type lateral ankle sprain. This is found in about 20% of patients with lateral ankle instability (This figure was created by the authors to be used in this paper).

The most common mechanism of acute ankle injury is a combination of inversion (Figure 2) and internal rotation forces associated with a plantar flexed or less frequently dorsiflexed foot [5]. The superior fascicle of ATFL is the weakest structure among the lateral ligaments of the ankle. It is the first ligament to be injured during ankle sprain [5,20,21]. In 65 to 80% of cases, there is an isolated rupture of the ATFL whereas a combined rupture of both ATFL and CFL (Figure 2) happens in around 20% of cases [5,20]. The PTFL is rarely injured during the abovementioned mechanism [21,22].

## 5. Diagnosis

Given that the purpose of this paper is to focus on the improvement of the postoperative management, the different aspects that should be addressed by anamnesis [7,23] and the clinical tests [5,7,23] that should be carried out in order to rule out other associated injuries with lateral ligamentous ruptures including osteochondral lesions, bone fractures and peroneal tendons lesions, will not be detailed in this article. Special tests are usually used to help surgeons diagnose ankle instability. Typically, Anterior Drawer (AD) and Talar Tilt (TT) tests are used to assess the integrity of ATFL and CFL, respectively [2,5,7,23]. However, Van Dijk et al. considered that the AD test may not be accurate because it applies isolated sagittal translation without taking into account the rotational laxity [24]. Phisitkul et al. described AnteroLateral Drawer (ALD) testing in which the foot is allowed to internally rotate while translating it anteriorly, and they showed that ALD is a more accurate test than AD in detecting ATFL inefficiency [25]. Frey et al. compared Magnetic Resonance Imaging (MRI) results with physical examination findings and found that grade II lesions were most often underestimated by clinicians with an accuracy of only 25% in diagnosing those lesions [26]. To clarify, a grade I injury involves ligament stretching with minimal swelling, grade II indicates a partial macroscopic tear, while grade III signifies a complete rupture of the ligaments [26]. Some authors have proposed delaying the physical examination in acute settings to improve the accuracy of testing [24].

## 6. Imaging

Classically, three imaging modalities have been used to help diagnose CLAI: stress radiographs, MRI and UltraSonography (US) [2]. While stress radiographs have been proposed as an objective tool to visualize the subjective findings of AD and TT tests, with established threshold values including 10 mm anterior translation of talus for AD testing, and 10° tilt for TT testing [27]. When performing comparative radiographs between the injured and the normal sides, a side-to-side difference of at least 5 mm for AD or at least 5° for TT is required to support the diagnosis of lateral ankle instability [2,27]. In 1993, Peyre and Rodineau showed that auto-varus active stress radiographs may provide better results compared with passive testing realized manually or using a stress device [28]. However, the variety of measurement methods make the results difficult to exploit and until today the main limitation of stress radiographs remains their low sensitivity, which is around 57% [2,5,7,23,28].

MRI is sensitive for identification of ligament defects and ruptures, but its static nature prevents its ability to assess ligament function [2,5,29]. In their retrospective study, Jolman et al. compared the MRI findings of 112 patients operated for CLAI with those of 75 patients referred for other pathologies. The authors identified a high sensitivity but a relatively low specificity (53.3%) and concluded that MRI can be useful to identify associated extra and intra-articular lesions that can be associated with CLAI rather than being a primary tool to help establish the diagnosis of CLAI [29].

Ultrasonography (US) has showed substantial accuracy with high sensitivity and specificity in detecting ATFL and CFL defects [2,5,30]. Dynamic US can be performed using stress maneuvers in order to assess lateral ankle stability in real time. Although favorable outcomes are being observed in some studies, further clinical and biomechanical studies are still necessary before validating it as a diagnostic tool for CLAI. Finally, useful data cannot be obtained with US unless it is performed by a skilled operator.

## 7. Surgical Treatment

**Figure 3:**
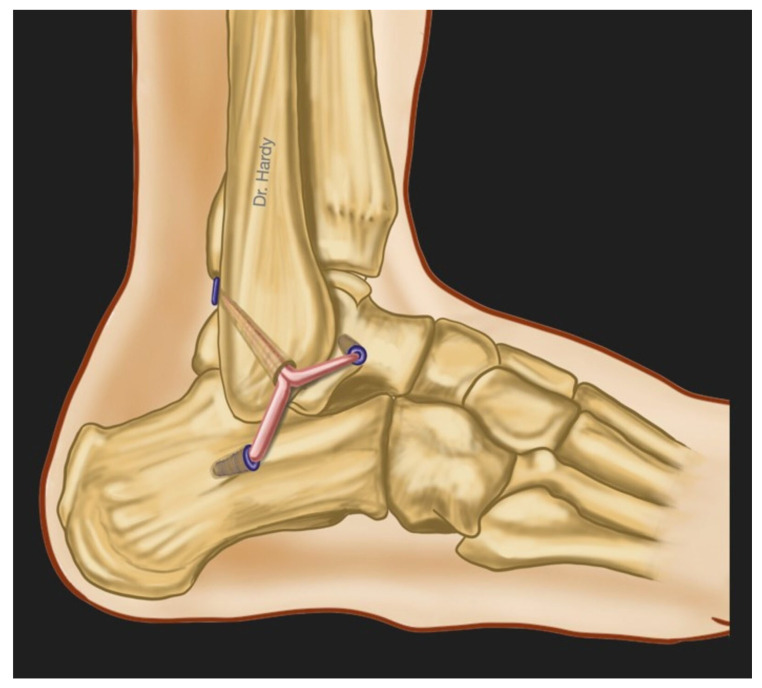
This figure represents the anatomic surgical reconstruction of the lateral ankle ligaments using an autograft: the gracilis tendon harvested from the patient’s knee (This figure was created by the authors to be used in this paper).

The surgical treatment of CLAI includes a number of procedures ranging from simple repair of the injured ligaments to reconstruction techniques. They can all be performed as open procedures or arthroscopically, and they can all be classified either as anatomic or non-anatomic techniques. Broström first described an anatomic open repair technique in 1966, consisting of tightening the ruptured lateral ligaments to the fibula [31]. Several modifications have been described subsequently, including the Gould et al. procedure that comprised an additional step, which is an augmentation of the repair using the inferior extensors retinaculum [32], then the Karlsson et al. modification that consisted of performing a shortening of the ligaments when they are elongated rather than disrupted [33]. The augmentation using non-absorbable suture-tape have been also advocated by some authors [5,34]. Non-anatomic reconstruction techniques involving tenodesis and including Chrisman-Snook, Evans and Watson-Jones procedures were initially introduced to replace the direct repair for cases with severely injured ligaments. However, they have been associated with many complications, including decreased ROM, recurrent lateral ankle instability and osteoarthritis. It was also shown that non-anatomic reconstruction may compromise the normal joint biomechanics [2,5,35]. Anatomic reconstruction procedures involve direct reconstruction of the ATFL and CFL using either autografts (Figure 3), allografts or synthetic ligaments. They have showed positive results and superior outcomes compared with non-anatomic reconstruction [2,5,36]. Nowadays, arthroscopically assisted and all-arthroscopic procedures are increasingly used for performing both repair and reconstruction with favorable outcomes in clinical studies. To our knowledge, we are still lacking high-level biomechanical evidence directly comparing arthroscopic versus open lateral ankle instability surgical techniques.

## 8. Postoperative Rehabilitation and Management

Postoperative rehabilitation is essential to achieve optimal functional outcomes without altering the stability of the ankle joint and increasing the risk of complications. However, we are still lacking uniform or optimal evidence-based rehabilitation protocols. In their systematic review including 14 articles with a total of 809 patients, Clements et al. showed that there is a wide variation of postoperative immobilization protocols as well as postoperative rehabilitation protocols [11]. The number of rehabilitation phases varied between 1 and 5, at times even differing between groups within the same study. Given the available literature findings, they suggested a four-phase protocol including progressive weight bearing, ROM, balance, proprioception and strengthening of peroneal muscles. The outcomes were measured based only on subjective functional assessments and objective outcome scores including, among others, the Foot and Ankle Ability Measure, the American Orthopedic Foot and Ankle Society questionnaire, the Karlsson Scoring, the Cumberland Ankle Instability Tool, and the Foot and Ankle Outcome Score.

In their systematic review including 1457 patients, Vopat et al. compared early and delayed postoperative mobilization protocols in patients after CLAI ligament repair surgery, and showed improved functional scores in early mobilization (EM) groups [4]. However, an objective radiographic laxity and higher complication rates were found in these patients. In a randomized study where EM after ligaments repair was defined as 7 to 10 days of plaster followed by an ankle brace, Karlsson et al. showed better functional outcomes in this group of patients compared with the delayed mobilization group, without noticing negative effects of EM on ankle stability [37]. Moreover, many surgeons are increasingly using anatomic reconstruction techniques with immediate weight bearing allowed postoperatively but there continues to be some debate on how to protect the reconstructed ligament if deemed necessary [2]. In a systematic review performed by Hunt et al. about the RTS following lateral ankle ligament repair or reconstruction, it was shown that the literature is clearly deficient concerning the presence of a consistent timeline, with identification of a variety of metrics used for the measurement of outcomes, and those metrics were tracked in very few studies [3]. They mentioned that among 360 papers identified, only 5.5% detailed a return to play timeline. Furthermore, they observed that the studies generally reported aggregated mean time to RTS and that the variation of reported timelines depends on the injury patterns, the surgical technique, the type of sport, and the postoperative use of bracing. They concluded that there is still lack of a well-defined meaningful tool or protocol to assess the readiness and the ability of athletes to RTS.

Many authors proposed the use of MRI as a potential useful tool for the follow-up of CLAI patients postoperatively since the signal intensity reflects the water content of the tissue, enabling the surgeons to track the graft healing process over time [16]. In order to increase the MRI effectiveness and reliability, some studies defined another MRI parameter taking into account the background signal in addition to signal intensity. This parameter is the signal to noise quotient (SNQ). It was initially used for graft maturity assessment in patients after anterior cruciate ligament (ACL) reconstruction. In fact, it has been demonstrated the presence of three stages of graft healing after ACL reconstruction: the early healing phase, then the proliferation phase, followed by the ligamentization or maturation final stage [13]. In a systematic review performed to evaluate the possible relationships between histologic findings, SNQ and clinical outcomes in graft healing assessment after ACL reconstruction using autograft, Van Groningen et al. concluded that the MRI SNQ does not predict either graft maturity or functional and clinical outcomes after ACL reconstruction [13]. Furthermore, they identified the presence of a heterogeneity of MRI methods used in the literature in addition to many technical restrictions.

According to all the aforementioned findings, we can consider that we are lacking meaningful, reliable and accurate modalities and methods that could help the surgeons improve the postoperative management of CLAI patients. Given the wide variety of the injury patterns and the surgical techniques described in the literature, in addition to the interindividual differences in the pain perception, we believe that we need to use objective and innovative outcome measurement modalities that may reflect more precisely the histological changes of tissue to track the healing process of the repaired/reconstructed ligaments and/or the mechanical stability of the ligaments and the ankle joint.

## 9. Alternative Outcome Monitoring Methods in Patients after CLAI Surgery

Bioelectrical impedance (BEImp), mechanical impedance (MImp) and near-infrared spectroscopy (NIRS) are three non-invasive measurement methods that can be used to improve the postoperative management of CLAI patients.

## 10. Bioelectrical Impedance Spectroscopy Measurement

**Figure 4:**
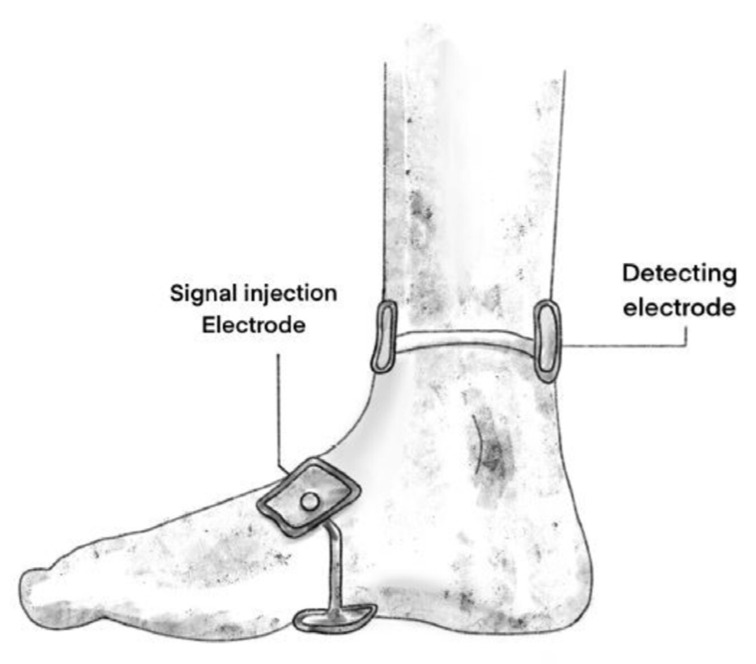
Bioelectrical impedance of the ankle ligaments can be measured by injecting an electrical current using an injection electrode and detecting the resultant voltage decrease across the biological sample via another electrode. The two electrodes are placed on either side of the joint (This figure was created by the authors to be used in this paper).

Bioelectrical impedance (BEImp) is a means for the assessment of the biological tissue composition by injecting an electrical current at defined frequencies and measuring the resultant voltage decrease across the biological sample (Figure 4). Impedance is a combination of two parameters: resistance and reactance. The former is the extent to which the current is limited when passing across the tested tissue while the latter represents the non-resistive imaginary component of impedance in an alternative current (AC) resulting from the effect of inductance or capacitance or both [38]. BEImp has been used in many medical fields, and we can find a recent increase in published studies evaluating its usefulness in assessment of tendon, ligament and joint pathologies. Mabrouk et al. tracked ankle edema using BEImp [38]. They initially designed a wearable measurement system with a specific algorithm that allowed the mapping of the outputted raw values generated by an impedance analyzer into accurate real and imaginary impedance values. In order to allow this mapping to be more accurate through the range of the measured frequencies, they used the standard 2R1C electrical circuit model to represent the biological tissue. Given the frequency dependency of the penetration depth of current in tissue, they realized a differential measurement technique by moving the ankle through multiple positions. This method performed on cadaver models as well as on a cohort of patients showed favorable outcomes with low interindividual and intra-individual variability. In an experimental study on rabbits, Yoon et al. demonstrated that BEImp could be a useful modality to make the diagnosis of tendinitis [39]. They used the dissipation factor (D) to increase their results accuracy. This factor is defined as the resistance to reactance ratio (D = resistance/reactance), and it seems to be a very meaningful index because its standard deviation is one-tenth that of the resistance and it also reduces the interface problems that happen between the electrodes and tissues.

## 11. Mechanical Impedance Measurement

**Figure 5:**
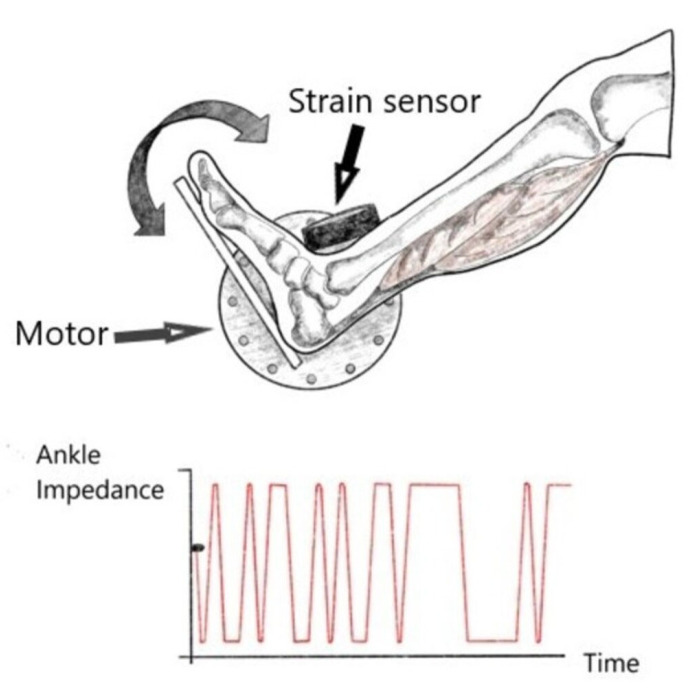
Mechanical impedance represents the dynamic relationship between imposed deformations or motions (through the rotatory motor as shown in this figure) and the resulting torques determined by using a strain sensor (This figure was created by the authors to be used in this paper).

Mechanical impedance (MImp) represents the dynamic relationship between imposed deformations or motions and the resulting torques [40]. The estimates of MImp of a joint allow for the quantification of the mechanical properties of the tested joint (Figure 5). Given that the biomechanics of a joint are determined by the mechanical properties of the muscles and tendons spanning it, MImp could be a substantial modality to track the healing process and graft maturity in patients after CLAI surgery as well as the progression of mechanical stability and potential early postoperative laxity over time. Although published studies classically measure the global joint MImp without taking into account the specific contribution from the tendon and muscle, there is actually recent evidence in the literature which supports our belief that MImp measurement may play an important role in the improvement of postoperative management by helping surgeons to assess the readiness of patients and athletes to RTS. Jakubowski et al. developed an experimental model and analysis methods to quantify tendon, muscle and joint impedance by using an actuator with a single degree of freedom to impose pseudorandom rotations to the ankle while the corresponding torques were being measured [41]. At the same time, the displacement of the medial gastrocnemius muscle–tendon junction was measured using B-mode ultrasound. From these measurements, they were able to estimate the correspondent impedance values in addition to the assessment of the muscle and tendon stiffness. This experiment holds substantial clinical significance as it established the credibility and consistency of a novel methodology for assessing in vivo muscle and tendon impedance during static postures and dynamic movements [41]. This approach enables the monitoring of fluctuations in muscle and tendon stiffness over time, opening avenues to delve into the mechanics governing posture and movement control. This is pivotal not only in enhancing our comprehension of unimpaired motor control but also in identifying and addressing impairments due to pathological changes [41]. By pinpointing the source of impairment, this technique facilitates targeted rehabilitation strategies. Furthermore, understanding the distinct roles of muscles and tendons is crucial for optimizing exoskeleton design and control, potentially enhancing walking efficiency by optimizing the muscle and tendon mechanics through the exoskeleton controller [41].

## 12. Near-Infrared Spectroscopy (NIRS)

**Figure 6:**
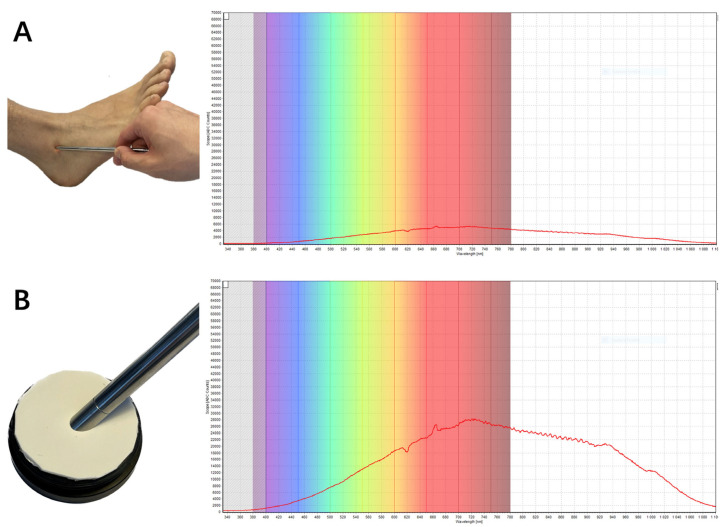
(**A**) Reflectance measurement of the Anterior TaloFibular Ligament (ATFL) of the ankle by using an optical fiber (Left). Inside this fiber, there are light transmitting and detecting fibers. The reflected light is converted into an electrical signal and visualized using a specific software (Right) (in this example, we used AvaSoft Version 8.14.0.0 to visualize the reflectance of ATFL with respect to wavelength). (**B**) White reference is needed when converting hyperspectral data into reflectance.

**Figure 7:**
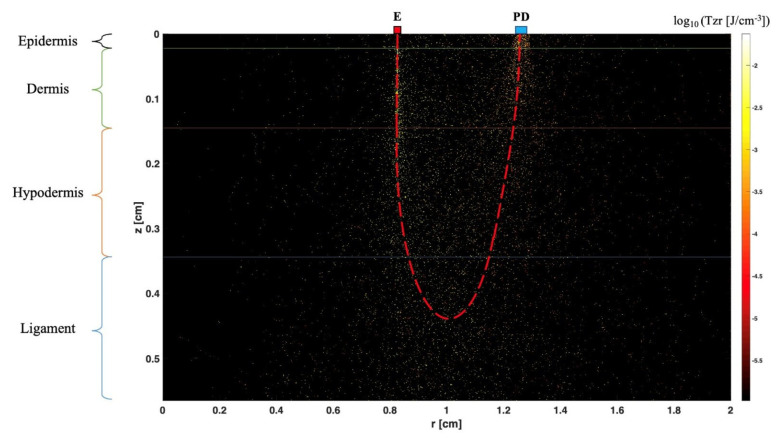
This figure represents a simulation realized by the authors of this paper using MCML software. It shows the penetration depth (z) of the light through the different layers of the ankle before reaching the ligament. In this example, we chose the distance between the light-emitting source (λ = 1300 nm) and the photodetector to be 0.4 cm (the positions of E and PD correspond to r = 0.8 cm and r = 1.2 cm, respectively). For r = 0.4 cm, the light penetration depth seems to be good enough to explore the reflectance pattern of the lateral ankle ligaments.

In 1977, F.F. Jöbsis first described the non-invasive in vivo application of NIRS by measuring the tissue oxygenation of tissue in real time [42]. His work was based on the principles of optical spectrophotometry. Technically, NIRS includes a light source defined as a light-emitting diode (LED) and/or a laser diode, which covers the near-infrared (NIR) range of wavelengths between 650 nm and 1000 nm, and a detector. Flexible optic fibers are generally used in this method in order to transmit the light from the source and to the detectors (Figure 6). When NIR passes through a tissue, the light can be absorbed, reflected, scattered and transmitted. In fact, these physical phenomena occur simultaneously but at different extents depending on the emitted wavelength, the nature and heterogeneity of the tested tissue among other factors. Furthermore, NIRS can be used in transmission mode, reflection mode or both depending on the degree of transparency of the tested sample to NIR. It should be mentioned that NIRS has been used in many medical and biological fields, including tissue oxygenation monitoring (central nervous system and somatic tissue), biological fluids and solutions composition analysis in addition to molecular structure and interaction analysis. Recent literature shows an increase in published studies describing the use of NIRS to characterize the optical parameters of human tissue, including fibrous tissue like ligaments and tendons, and to estimate their mechanical properties [43,44]. In an experimental study, Torniainen et al. showed that NIRS can estimate the biomechanical properties of knee ligaments and patellar tendons [44]. In another experimental study of a rat induced-knee osteoarthritis model, Afara et al. demonstrated that NIRS data and spectral feedbacks enabled the separation of the cartilage samples relative to the severity of osteoarthritis with significant correlation between NIRS findings and the histologic Mankin score [45]. Finally, it should be noted that researchers can use sophisticated software that can allow tissue modeling and simulations performance that could help them estimate the possible pathways and penetration depth of the emitted photons [46]. Thus, this makes it possible, for example, to determine the ideal distance between light sources and detectors as well as the penetration depth of the light before the application of the technique on ex vivo and in vivo models. As shown in Figure 7, the authors of this paper realized a simulation utilizing MCML software and employing light of specific wavelength λ = 1300 nm. A distance of 4 mm between the light source and the photodetector demonstrated adequacy in enabling comprehensive exploration of the reflectance pattern of the lateral ankle ligaments, allowed by substantial light penetration depth. The assessment of 12 normal ankles revealed a consistent reflectance curve pattern, albeit with minor discrepancies in curve amplitudes attributed to variations in reflected photon count, influenced by differences in skin color and ethnicity. These preliminary observations necessitate validation through extensive clinical studies encompassing larger cohorts, comprising individuals with both normal and pathologic ankles. These findings hold promise for advancing the evaluation of lateral ankle ligaments, with significant implications for their validation. Furthermore, leveraging these insights in subsequent research endeavors could inform the development of devices aimed at pre- and post-surgical assessment of ankle ligaments by manufacturers.

## 13. Summary

1. Ankle sprain represents a frequent pathology among athletes and the general population. Inversion-type is the most common mechanism of injury. Patients that do not respond to conservative management may develop CLAI.
2. CLAI is diagnosed following a thorough anamnesis, physical examination and medical imaging including stress radiographs, MRI and ultrasonography. AD testing should be replaced by ALD testing in clinical practice because it allows detection of both anteroposterior and rotational talar instability. Stress radiographs are limited by their low sensitivity. MRI may be better used to rule out injuries that could be associated with CLAI. Dynamic US is showing good outcomes in recent published studies, but these results are yet to be validated.
3. There is a wide variety of surgical procedures that address CLAI, ranging from simple repairs to more complex reconstructions. Besides non-anatomic procedures, which are increasingly abandoned due to their association with alteration of normal joint biomechanics, there is no superiority demonstrated of one technique over the others if the potential contraindications are taken into account.
4. The ideal postoperative management remains unknown. There is still a lack of accurate outcome assessment methods which can reflect the healing process’s histological stage and the mechanical stability of the repaired or reconstructed ligament. In most of the published studies regarding rehabilitation protocols and management of patients after CLAI surgery, the outcomes are being measured using a wide range of functional assessment scores and stress radiographs with the absence of meaningful, precise, reliable and uniform methods to assess those outcomes.
5. MRI is limited by its static character, and it seems that it cannot be a useful tool for postoperative management of patients after CLAI surgery because recent evidence showed that SNQ failed to predict the histological phase of the repaired or reconstructed healing ligament.
6. The integration of BEImp, MImp and NIRS into clinical practice holds immense promise for CLAI patients. These non-invasive and robust measurement methods, widely established across medical domains, show considerable potential in assessing ligament, tendon, cartilage, and joint pathologies. While existing literature hints at their effectiveness, comprehensive studies are imperative to confirm their individual and combined accuracy. Validating these modalities could establish them as pivotal tools for surgeons, providing precise biomechanical data and enabling the objective assessment of lateral ankle ligaments’ stiffness, maturation, and function postoperatively. Moreover, these technologies extend their utility beyond surgical scenarios; they could be valuable in evaluating patients undergoing medical management and even serve diagnostic purposes. Envisioning these advancements within a wearable, easily portable device not only enhances their clinical utility but also empowers physicians to employ objective measurement tools for comprehensive assessment and management of CLAI patients. This departure from exclusive reliance on subjective measurement tools, such as Patient-Related Outcome Measures (PROMs), signifies a substantial stride toward bolstering the precision and objectivity of assessments in clinical practice.

## Data Availability

No new data were created or analyzed in this study. Data sharing is not applicable to this article.

## References

[1] Drakos M., Hansen O., Kukadia S. (2022). Ankle Instability. Foot Ankle Clin..

[2] Chang S.H., Morris B.L., Saengsin J., Tourné Y., Guillo S., Guss D., DiGiovanni C.W. (2021). Diagnosis and Treatment of Chronic Lateral Ankle Instability: Review of Our Biomechanical Evidence. J. Am. Acad. Orthop. Surg..

[3] Hunt K.J., Fuld R.S., Sutphin B.S., Pereira H., D’Hooghe P. (2017). Return to sport following lateral ankle ligament repair is under-reported: A systematic review. J. ISAKOS Jt. Disord. Orthop. Sports Med..

[4] Vopat M.L., Tarakemeh A., Morris B., Hassan M., Garvin P., Zackula R., Mullen S., Schroeppel P., Vopat B.G. (2019). Early versus Delayed Mobilization Post-Operative Protocols for Primary Lateral Ankle Ligament Repair: A Systematic Review and Meta-analysis. Foot Ankle Orthop..

[5] Machado M., Amado P., Babulal J. (2021). Ankle instability—Review and new trends. J. Orthop. Trauma Rehabil..

[6] Bestwick-Stevenson T., Wyatt L.A., Palmer D., Ching A., Kerslake R., Coffey F., Batt M.E., Scammell B.E. (2021). Incidence and risk factors for poor ankle functional recovery, and the development and progression of posttraumatic ankle osteoarthritis after significant ankle ligament injury (SALI): The SALI cohort study protocol. BMC Musculoskelet. Disord..

[7] Tourné Y., Besse J.-L., Mabit C. (2010). Chronic ankle instability. Which tests to assess the lesions? Which therapeutic options?. Orthop. Traumatol. Surg. Res..

[8] Dromzée E., Granger B., Rousseau R., Steltzlen C., Stolz H., Khiami F. (2019). Long-Term Results for Treatment of Chronic Ankle Instability with Fibular Periosteum Ligamentoplasty and Extensor Retinaculum Flap. J. Foot Ankle Surg..

[9] Cho B.-K., Kim Y.-M., Shon H.-C., Park K.-J., Cha J.-K., Ha Y.-W. (2015). A Ligament Reattachment Technique for High-Demand Athletes with Chronic Ankle Instability. J. Foot Ankle Surg..

[10] Porter D.A., Kamman K.A. (2018). Chronic Lateral Ankle Instability. Foot Ankle Clin..

[11] Clements A.D., Belilos E.B., Keeling L., Kelly M., Casscells N. (2021). Postoperative Rehabilitation of Chronic Lateral Ankle Instability: A Systematic Review. Sports Med. Arthrosc. Rev..

[12] Faure C., Deplus F., Besse J.L., Moyen B., Bochu M. (1997). Chronic external instability of the ankle. Contribution of dynamic radiographies, x-ray computed tomography and x-ray computed tomographic arthrography. J. Radiol..

[13] van Groningen B., van der Steen M.C., Janssen D.M., van Rhijn L.W., van der Linden A.N., Janssen R.P.A. (2020). Assessment of Graft Maturity After Anterior Cruciate Ligament Reconstruction Using Autografts: A Systematic Review of Biopsy and Magnetic Resonance Imaging studies. Arthrosc. Sports Med. Rehabil..

[14] Schroeder J., Barzilay Y., Hasharoni A., Kaplan L. (2011). Long-term outcome of surgical correction of congenital kyphosis in patients with myelomeningocele (MMC) with segmental spino-pelvic fixation. Evid. Based Spine-Care J..

[15] Vega J., Malagelada F., Céspedes M.-C.M., Dalmau-Pastor M. (2020). The lateral fibulotalocalcaneal ligament complex: An ankle stabilizing isometric structure. Knee Surg. Sports Traumatol. Arthrosc..

[16] Umans H., Cerezal L., Linklater J., Fritz J. (2022). Postoperative MRI of the Ankle and Foot. Magn. Reson. Imaging Clin. North Am..

[17] Brantigan J., Pedegana L., Lippert F. (1977). Instability of the subtalar joint. Diagnosis by stress tomography in three cases. Minerva Anestesiol..

[18] Tochigi Y., Amendola A., Rudert M.J., Baer T.E., Brown T.D., Hillis S.L., Saltzman C.L. (2004). The role of the interosseous talocalcaneal ligament in subtalar joint stability. Foot Ankle Int..

[19] Ozeki S., Yasuda K., Kaneda K., Yamakoshi K., Yamanoi T. (2002). Simultaneous strain measurement with determination of a zero strain reference for the medial and lateral ligaments of the ankle. Foot Ankle Int..

[20] Hintermann B., Boss A., Schäfer D. (2002). Arthroscopic Findings in Patients with Chronic Ankle Instability. Am. J. Sports Med..

[21] Vega J., Allmendinger J., Malagelada F., Guelfi M., Dalmau-Pastor M. (2020). Combined arthroscopic all-inside repair of lateral and medial ankle ligaments is an effective treatment for rotational ankle instability. Knee Surg. Sports Traumatol. Arthrosc..

[22] Vega J., Peña F., Golanó P. (2014). Minor or occult ankle instability as a cause of anterolateral pain after ankle sprain. Knee Surg. Sports Traumatol. Arthrosc..

[23] Guillo S., Bauer T., Lee J., Takao M., Kong S., Stone J., Mangone P., Molloy A., Perera A., Pearce C. (2013). Consensus in chronic ankle instability: Aetiology, assessment, surgical indications and place for arthroscopy. Orthop. Traumatol. Surg. Res..

[24] van Dijk C.N., Lim L.S.L., Bossuyt P.M.M., Marti R.K. (1996). Physical examination is sufficient for the diagnosis of sprained ankles. J. Bone Jt. Surg..

[25] Phisitkul P., Chaichankul C., Sripongsai R., Prasitdamrong I., Tengtrakulcharoen P., Suarchawaratana S. (2009). Accuracy of Anterolateral Drawer Test in Lateral Ankle Instability: A Cadaveric Study. Foot Ankle Int..

[26] Frey C., Bell J., Teresi L., Kerr R., Feder K. (1996). A Comparison of MRI and Clinical Examination of Acute Lateral Ankle Sprains. Foot Ankle Int..

[27] Hoffman E., Paller D., Koruprolu S., Drakos M., Behrens S.B., Crisco J.J., DiGiovanni C.W. (2011). Accuracy of Plain Radiographs Versus 3D Analysis of Ankle Stress Test. Foot Ankle Int..

[28] Peyre M., Rodineau J. (1993). L’auto Varus: Une Technique D’exploration des Instabilités Externes de Cheville.

[29] Jolman S., Robbins J., Lewis L., Wilkes M., Ryan P. (2017). Comparison of Magnetic Resonance Imaging and Stress Radiographs in the Evaluation of Chronic Lateral Ankle Instability. Foot Ankle Int..

[30] Alshalawi S., Galhoum A.E., Alrashidi Y., Wiewiorski M., Herrera M., Barg A., Valderrabano V. (2018). Medial Ankle Instability. Foot Ankle Clin..

[31] Broström L. (1966). Sprained ankles. VI. Surgical treatment of “chronic” ligament ruptures. Acta Chir Scand.

[32] Gould N., Seligson D., Gassman J. (1980). Early and Late Repair of Lateral Ligament of the Ankle. Foot Ankle.

[33] Karlsson J., Bergsten T., Lansinger O., Peterson L. (1989). Surgical treatment of chronic lateral instability of the ankle joint. Am. J. Sports Med..

[34] Viens N.A., Wijdicks C.A., Campbell K.J., LaPrade R.F., Clanton T.O. (2014). Anterior Talofibular Ligament Ruptures, Part 1. Am. J. Sports Med..

[35] Hunt K.J., Pereira H., Kelley J., Anderson N., Fuld R., Baldini T., Kumparatana P., D’hooghe P. (2019). The Role of Calcaneofibular Ligament Injury in Ankle Instability: Implications for Surgical Management. Am. J. Sports Med..

[36] Camacho L.D., Roward Z.T., Deng Y., Latt L.D. (2019). Surgical Management of Lateral Ankle Instability in Athletes. J. Athl. Train..

[37] Karlsson J., Rudholm O., Bergsten T., Faxen E., Styf J. (1995). Early range of motion training after ligament reconstruction of the ankle joint. Knee Surg. Sports Traumatol. Arthrosc..

[38] Mabrouk S., Hersek S., Jeong H.K., Whittingslow D., Ganti V.G., Wolkoff P., Inan O.T. (2020). Robust Longitudinal Ankle Edema Assessment Using Wearable Bioimpedance Spectroscopy. IEEE Trans. Biomed. Eng..

[39] Yoon K., Lee K.W., Kim S.B., Han T.R., Jung D.K., Roh M.S., Lee J.H. (2010). Electrical impedance spectroscopy and diagnosis of tendinitis. Physiol. Meas..

[40] Ludvig D., Whitmore M.W., Perreault E.J. (2022). Leveraging Joint Mechanics Simplifies the Neural Control of Movement. Front. Integr. Neurosci..

[41] Jakubowski K.L., Ludvig D., Bujnowski D., Lee S.S.M., Perreault E.J. (2022). Simultaneous Quantification of Ankle, Muscle, and Tendon Impedance in Humans. IEEE Trans. Biomed. Eng..

[42] Jöbsis F.F. (1977). Noninvasive, Infrared Monitoring of Cerebral and Myocardial Oxygen Sufficiency and Circulatory Parameters. Science.

[43] Fan C., Shuaib A., Yao G. (2011). Path-length resolved reflectance in tendon and muscle. Opt. Express.

[44] Torniainen J., Ristaniemi A., Sarin J.K., Prakash M., Afara I.O., Finnilä M.A.J., Stenroth L., Korhonen R.K., Töyräs J. (2022). Near infrared spectroscopic evaluation of biochemical and crimp properties of knee joint ligaments and patellar tendon. PLoS ONE.

[45] Afara I., Prasadam I., Crawford R., Xiao Y., Oloyede A. (2012). Non-destructive evaluation of articular cartilage defects using near-infrared (NIR) spectroscopy in osteoarthritic rat models and its direct relation to Mankin score. Osteoarthr. Cartil..

[46] Wang L., Jacques S.L., Zheng L. (1997). Conv—Convolution for responses to a finite diameter photon beam incident on multi-layered tissues. Comput. Methods Programs Biomed..

